# CONTACT-FREE SPORTS ACTIVITIES DURING COVID-19: IMPACT ON SUBJECTIVE HEALTH AND STAMINA AMONG KOREAN OLDER ADULTS

**DOI:** 10.1093/geroni/igad104.3050

**Published:** 2023-12-21

**Authors:** Gaeun Han, Juhyeong Lee, Giyeon Kim

**Affiliations:** Chung-Ang University, Seoul, Republic of Korea; Chung-Ang University, Seoul, Republic of Korea; Chung-Ang University, Seoul, Republic of Korea

## Abstract

Despite the importance of participation in sports activities among older adults, COVID-19 pandemic has limited access to sports facilities, which may threaten the physical and mental health of older adults. This study examines whether the relationship between age and subjective health and stamina is moderated by participation in contact-free sports activities among Korean older adults. A nationally representative sample of Korean older adults aged 65 or older and doing regular exercise (N=1,118) was drawn from the 2021 National Survey on Sports for All. A moderation analysis was conducted by PROCESS macro using SPSS 26.0. Results showed that age was negatively associated with older adults’ subjective health (B = -.0314, p < .001) and subjective stamina (B = -.0259, p < .001), respectively. Participation in contact-free sports activities moderated the relationship between age and subjective health (B = .0266, p < .05) and subjective stamina (B= .0364, p < .01), indicating that the effect of age on subjective health/stamina varies by participation in contact-free sports activities. However, the conditional effect of age on older adults’ subjective health and stamina was significant only for those not participating in contact-free sports activities. The findings suggest that contact-free sports activities may mitigate the association between aging and physical decline in later life, especially during the pandemic and play a vital role in maintaining health and well-being of older adults.

